# Rice Cinnamoyl CoA Reductase-like Gene *OsCCR14* Involved in Heat Stress via Regulation Lignin and Flavonoid Accumulation

**DOI:** 10.3390/plants14233626

**Published:** 2025-11-28

**Authors:** Hongwei Wang, Wei Tian, Yulu Teng, Yuxin Xue, Simin Qin, Jiaxin Liu, Shuangcheng Ding

**Affiliations:** 1 MARA Key Laboratory of Sustainable Crop Production in the Middle Reaches of the Yangtze River (Co-Construction by Ministry and Province), Yangtze University, Jingzhou 434025, China; wanghw@yangtzeu.edu.cn; 2 Hubei Key Laboratory of Food Crop Germplasm and Genetic Improvement, Wuhan 430064, China; 3 Agricultural College, Yangtze University, Jingzhou 434000, China

**Keywords:** CCR-like protein, thermotolerance, lignin level, flavonoid metabolism

## Abstract

Cinnamoyl-CoA reductases (CCRs) and their homologs, CCR-like proteins, play key roles in plant secondary metabolism and defense against environmental stresses; however, their functions in heat stress responses remain unclear. In this study, phylogenetic and sequence alignment analyses revealed that OsCCR14 encodes a CCR-like protein. qRT-PCR assays showed that OsCCR14 is predominantly expressed in roots and is induced by heat stress. Notably, overexpression of OsCCR14 increased lignin content, and transgenic plants with enhanced OsCCR14 expression exhibited higher flavonoid accumulation in roots. Conversely, knockout of OsCCR14 reduced flavonoid content and impaired seedling heat tolerance. Furthermore, OsCCR14 overexpression improved heat tolerance, accompanied by increased root lignification and flavonoid accumulation. These results indicate that OsCCR14 acts as a critical regulator of lignin and flavonoid metabolism, thereby providing a potential target for enhancing crop heat tolerance.

## 1. Introduction

Rice (*Oryza sativa* L.), which serves as a staple food for nearly half of the global population, frequently suffers from various environmental stress induced by climate warming. High-temperature (HT) stress, which has emerged as a major threat to rice production, occurs when temperatures rise 5 °C above rice’s optimal growth range and trigger a series of cellular and metabolic responses in the plant to sustain its survival and growth [1]. HT stress adversely affects rice growth across all developmental stages, spanning from germination and seedling establishment to anthesis, grain filling, and grain maturation [2,3]. Notably, it has been documented that each 1 °C increase in global temperature leads to a 3.2% reduction in rice yield [4]. Thus, enhancing stress resilience in rice has become an imperative strategy to protect global agricultural productivity.

HT stress primarily damages plants by disrupting their metabolic homeostasis, which leads to the excessive accumulation of reactive oxygen species (ROS) free radicals [5,6]. While at physiological levels ROS function as signaling molecules that regulate plant adaptation to various stresses, excessive ROS production often inflicts severe harm on plant cells [7]. Thus, maintaining strict control over ROS homeostasis is essential for plant survival. Numerous studies have demonstrated that enhanced antioxidant enzyme activity contributes to improved plant heat tolerance [8]. Beyond antioxidant systems, secondary metabolites also play a crucial role in enabling plants to adapt to adverse environmental conditions, including temperature stress [9,10]. Lignin is an important polymeric phenylpropanoid derivative that is mainly deposited in the secondary cell wall to maintain cell wall integrity during plant heat stress responses [11]. Formation of the lignin polymer requires three core monolignols, p-coumaryl alcohol, coniferyl alcohol, and sinapyl alcohol. The synthesis of these monolignols originates from phenylalanine and was catalyzed by some enzymes, including phenylalanine ammonia lyase (PAL), cinnamic acid 4-hydroxylase (C4H), and 4-coumarate-CoA ligase (4CL), driving early reactions to produce common phenylpropanoid intermediates [12,13,14]. These intermediates are directed to the monolignol-specific pathway for lignin production, they can also diverge into flavonoid-specific pathways (e.g., via chalcone synthase, CHS) to synthesize flavonoids. Flavonoids act as antioxidants, with the ability to reduce oxidative damage that arises from ROS accumulation; this accumulation is induced by extreme temperatures [15,16,17]. Therefore, changes in the activity and gene expression levels of phenylpropanoid pathway affect the synthesis of plant secondary metabolites, which exerts a crucial role in enhancing the capacity of plants to deal with high-temperature stress [18,19]. Although the flavonoid biosynthetic pathway and lignin biosynthetic pathway have been extensively investigated, the relationship between flavonoid biosynthesis and lignin biosynthesis within the plant phenylpropanoid pathway, along with how they are regulated and how they respond to high-temperature stress, remains unclear.

CCRs, the first enzyme in the monolignol-specific branch of the phenylpropanoid pathway, catalyze the conversion of cinnamoyl-CoA into their corresponding cinnamaldehydes [20,21]. To date, *CCR* genes have been characterized in a wide range of plant species [22,23,24,25,26,27]. They are highly diverse, encompassing members classified as genuine CCRs and CCR-like genes. In rice, it has been identified as 33 CCRs and CCR-like genes. For instance, OsCCR1, as an OsRac1 effector and lignin biosynthetic enzyme, participates in defense signaling [28]. *OsCCR10* overexpression improves drought tolerance during vegetative growth stages, whereas *OsCCR10* knock-out mutants mediated by CRISPR/Cas9 displayed decreased root lignin accumulation and substantially decreased drought tolerance compared to WT controls [29]. A NWYCY motif is recognized as a signature motif of CCR, distinguishing genuine CCRs from CCR-like proteins due to its significant impact on CCR catalytic activity. Another more reliable classification feature is the novel H202(X)2K205 motif (H-X-X-K, CCR-SBM or substrate binding motif), reported by Chao et al. [30]. Like CCRs, CCR-like genes belong to the short-chain dehydrogenase/reductases (SDR) superfamily, with encoded proteins containing common motifs (e.g., catalytic triads, NADPH-binding motifs), but they lack activity in converting cinnamoyl-CoA esters to hydroxy-cinnamaldehydes [25], and their functions remain unclear.

Previous transcriptome analysis revealed a complex network of gene expression changes under heat stress [31]. Subsequently, it was observed that Os09g31514, previously identified as a CCR-like gene and designated as *OsCCR14* [25], exhibited upregulated expression patterns in heat-tolerant genotyping cultivator rice plants, suggesting an important role in heat stress response. However, the specific role of this gene in regulating phenylpropanoid metabolism and its impact on heat tolerance remained unclear. Notably, Borah et al. proposed that this specific gene might encode a dihydroflavonol-4-reductase, a function distinct from the canonical CCR activity [32]. Therefore, in this study, *OsCCR14* was cloned and analyzed to determine its phylogenetic relationship. Additionally, the pattern of *OsCCR14* gene expression in response to heat stress and various hormone treatments, and its protein subcellular localization were analyzed. Importantly, ectopic expression of *OsCCR14* in Arabidopsis significantly increases lignin and flavonoid content, enhancing plant heat tolerance. The heat tolerance of *osccr14* mutants and *OsCCR14* overexpression lines at seedling stage was also evaluated in rice, revealing that *OsCCR14* positively regulated heat tolerance. The findings from this study would provide valuable insights into the molecular mechanisms underlying heat tolerance in rice and help to identify potential targets for crop improvement.

## 2. Results

### 2.1. Sequence and Phylogenetic Analysis of OsCCR14

**Figure 1:**
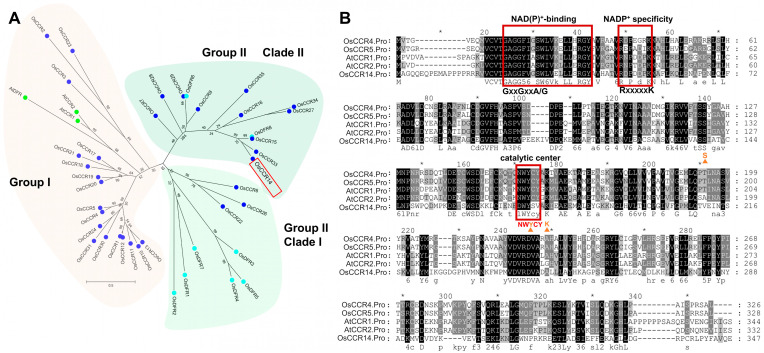
Phylogenetic and sequence analyses of OsCCR14. (**A**) Phylogenetic relationships of CCRs and DFRs from *Oryza sativa* and *Arabidopsis thaliana*. The evolutionary history was inferred by using the Maximum Likelihood method based on the JTT matrix-based model. Branch support was evaluated via 1000 bootstrap replicates, and bootstrap values are indicated at each branch node. The species name and the accession number of sequences used to construct the phylogenetic tree are listed in Table S1. (**B**) Alignment of OsCCR14 and other CCR proteins. The conserved motifs are indicated with a red box and the catalytic triad Ser–Tyr–Lys is indicated with an orange triangle. In sequence alignment, asterisks (*) serve as a visual annotation to indicate the length ranges of the aligned sequences.

*OsCCR14* was previously identified as a gene encoding cinnamoyl CoA reductases; it uses NAD(P)(H) as a co-factor for catalysis and belongs to SDR superfamily [25]. Given that DFR and CCR are proposed to share a common ancestral origin, a phylogenetic tree was constructed with a focus on rice-derived sequences. This tree included two Arabidopsis *CCR* genes (*AtCCRs*), thirty-two rice *CCR* genes (*OsCCRs*), and nine putative rice and Arabidopsis *DFR* genes (eight *OsDFRs* and one *AtDFR1*) [33]. Phylogenetic analysis revealed two distinct monophyletic groups: Group I and Group II (Figure 1A). Group I encompasses the majority of *CCR* genes, including the Arabidopsis homologs *AtCCR1* and *AtCCR2*. Group II is further subdivided into two well-supported clades, each containing a mixture of *OsDFR* and *OsCCR* genes. Notably, Clade II comprises 11 *OsCCR* members (including *OsCCR14*) and two *OsDFR* genes (Figure 1A).

Then, the protein sequence of OsCCR14 was aligned with those of functionally characterized CCR proteins. Sequence alignment uncovered that the most prominent homology across all analyzed peptide sequences resides in the N-terminal NAD(P)-binding motif. This region includes three key conserved elements: an NADP^+^-binding motif harboring the signature G-X-X-G-X-X-A/G segment, an NADP^+^-specific motif containing the R-X-X-X-X-X-K sequence, and the Ser-Tyr-Lys catalytic triad—all of which are critical for SDR superfamily enzyme function (Figure 1B). However, a notable divergence was observed in the NWYCY motif: this signature sequence, which is indispensable for CCR catalytic activity, is replaced by DWYSV in OsCCR14, with amino acid mismatches at the 1st (asparagine [N] → aspartic acid [D]), 4th (cysteine [C] → serine [S]), and 5th (tyrosine [Y] → valine [V]) positions. Additionally, another CCR-specific signature motif, containing the H-X-X-K sequence that mediates binding to the highly electronegative region of CoA, exhibits complete sequence variation in OsCCR4, OsCCR5, and OsCCR14 (Figure 1B). Collectively, these bioinformatic analyses confirm that OsCCR14 might encode a CCR-like protein.

### 2.2. Expression Analysis of OsCCR14

**Figure 2:**
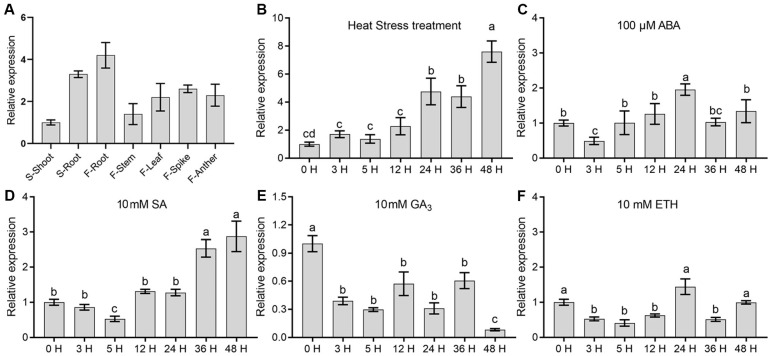
Expression profiles analysis of *OsCCR14.* (**A**) qRT-PCR analysis of *OsCCR14* in different tissues during different developmental stages. S and F stand for the seedling stage and flowering stage, respectively. (**B**) Expression of *OsCCR14* in response to 45 °C heat stress. (**C**) Expression of *OsCCR14* in response to 100 μM ABA. (**D**) Expression of *OsCCR14* in response to 10 mM SA. (**E**) Expression of *OsCCR14* in response to 10 mM GA_3_. (**F**) Expression of *OsCCR14* in response to 10 mM ETH (ethephon). *Tubulins* gene transcript level was used as an internal control for data normalization. Data were presented as mean ± SD, based on three independent biological replicates. Bars with different letters indicate statistically significant differences (*p* ≤ 0.05, ANOVA with Duncan’s test).

To gain insights into the potential biological function of OsCCR14, we first examined its spatiotemporal expression patterns in 3-week-old wild-type (Nipponbare) seedlings, as well as in plants at the flowering stage. Quantitative real-time PCR (qRT-PCR) results showed that at the seedling stage, *OsCCR14* transcripts accumulated relatively abundantly in roots, whereas expression levels in above-ground tissues were significantly lower (Figure 2A). At the flowering stage, *OsCCR14* also exhibited notably high expression in roots, followed by floral organs, with the lowest expression detected in stems (Figure 2A).

We further investigated *OsCCR14* expression in response to heat stress and treatments with various exogenous hormones. As shown in Figure 2B, high-temperature stress induced a dramatic upregulation of *OsCCR14* expression. With prolonged treatment duration, the expression fold change gradually increased, reaching ~4-fold and ~8-fold at 24 h and 48 h post-treatment, respectively. In contrast, *OsCCR14* showed only mild responsiveness to exogenous abscisic acid (ABA) and salicylic acid (SA) treatments. Relative to untreated control plants, its expression was elevated by approximately 2.0-fold under these two hormone treatments (Figure 2C,D). Notably, *OsCCR14* expression displayed a significant downregulation trend in response to gibberellic acid (GA_3_) treatment: expression levels decreased progressively, reaching a minimum at 48 h post-treatment with an approximate 11-fold downregulation compared to the control (Figure 2E). By comparison, *OsCCR14* exhibited only weak responsiveness to ethephon (Figure 2F). Collectively, these data suggests that *OsCCR14* genes may play important roles in rice development and abiotic stress responses, especially heat stress responses.

### 2.3. Subcellular Localization of OsCCR14

**Figure 3:**
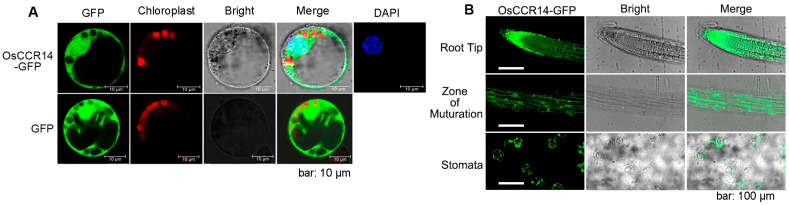
Subcellular and tissues localization of OsCCR14. (**A**) Subcellular localization of the OsCCR14-GFP fusion protein in rice cells. The *35S:GFP* and *35S:OsCCR14-GFP* constructs were transformed into rice leaf protoplasts. DAPI was used as a nuclear localization signal marker. (**B**) Root and stomatal localization of OsCCR14-GFP in *35S:OsCCR14-GFP* transgenic plants. Ten-day-old seedlings of *35S:OsCCR14-GFP* transgenic plants were observed under a fluorescence microscope.

To determine the subcellular localization of the OsCCR14 protein, we transiently expressed two constructs in rice protoplasts: a control vector with green fluorescent protein (GFP) driven by the enhanced *CaMV 35S* promoter, and a fusion construct (*35S:OsCCR14-GFP*) encoding OsCCR14 linked to GFP. Nuclei were stained with 4′,6-diamidino-2-phenylindole (DAPI) for visualization. The fluorescence signal of OsCCR14-GFP was detected in both the cytoplasm and nucleus (Figure 3A). Additionally, we analyzed stable transgenic *Arabidopsis thaliana* plants expressing *35S:OsCCR14-GFP*. In these plants, the OsCCR14-GFP fusion protein was predominantly observed in roots and guard cells (Figure 3B). Specifically, in the root tip and maturation zone of transgenic Arabidopsis, 35S:OsCCR14-GFP localized to both the cytoplasm and nucleus—consistent with the pattern observed in rice protoplasts.

### 2.4. Impact of OsCCR14-Overexpression on Lignin Content, Flavonol Fluorescence, and Heat Tolerance in Arabidopsis

**Figure 4:**
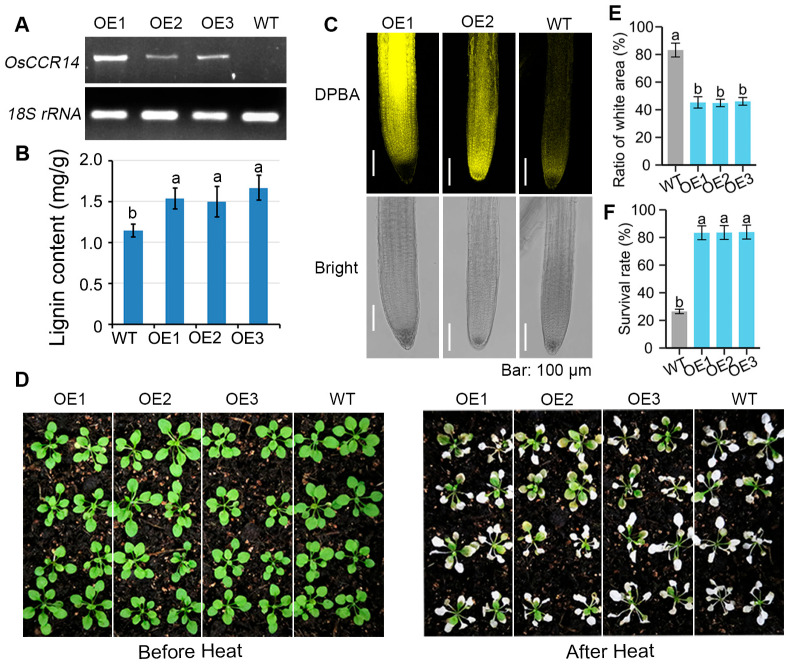
Analysis of lignin, flavonoid, and heat tolerance phenotypes in *OsCCR14*-transgenic Arabidopsis lines. (**A**) RT-PCR analysis of *OsCCR14* expression. (**B**) Lignin content quantified with the thioacidolysis method from 2-week-old transgenic plants. (**C**) DPBA staining showing flavonoid accumulation patterns in roots of 7-day-old seedlings. (**D**) Phenotype of *OsCCR14* overexpressing Arabidopsis thaliana lines under high temperature stress. (**E**) Proportion of white leaf area of *OsCCR14* overexpressing Arabidopsis lines under HT stress. (**F**) Survival rate of *OsCCR14* overexpressing Arabidopsis lines under HT stress. Values represented mean ± standard deviation of three independent biological replicates. Different letters above bars denoted significant differences (*p* ≤ 0.05, ANOVA with Duncan’s test). Colors are used solely to distinguish between different materials.

To further clarify the effect of *OsCCR14* expression level on lignin content, we analyzed the lignin level in *OsCCR14*-transgenic Arabidopsis lines. A construct containing the *OsCCR14* coding sequence driven by the *35S* promoter was generated and subsequently introduced into wild-type Arabidopsis thaliana (Col-0) via genetic transformation. RT-PCR analysis confirmed that OE1, OE2, and OE3 were representative overexpression lines, which were then used for subsequent experiments (Figure 4A). The impact of *OsCCR14* overexpression on lignin content was evaluated first. A statistically significant increase in lignin content was observed in OE1, OE2, and OE3 compared with the wild-type control, with an average of approximately 20% (Figure 4B). Considering the “metabolic flux competition” between lignin and flavonoids, we examined the effect of *OsCCR14* overexpression on root flavonol content by visualizing in vivo flavonol fluorescence. Seven-day-old seedlings were soaked in a solution containing diphenylboric acid-2-aminoethyl ester (DPBA), and flavonols were observed under a confocal microscope. Interestingly, a stronger fluorescence signal was detected in *OsCCR14* overexpression lines, with OE1 showing the most intense signal (Figure 4C).

Given that *OsCCR14* expression is induced by SA and high temperature (HT), and that *OsCCR14* promotes flavonoid accumulation, we hypothesized that *OsCCR14* contributes to plant responses to heat stress. To test this hypothesis, we compared the heat tolerance of *OsCCR14* overexpression lines (OE1, OE2, OE3) and wild-type Arabidopsis plants using 2-week-old seedlings grown under normal conditions. Seedlings were subjected to 42 °C heat treatment for 48 h, followed by a 7-day recovery period at 22 °C. As shown in Figure 4D, the survival rates of *OsCCR14* overexpression lines were consistently higher (ranging from 83.4% to 93.6%) compared to wild-type plants (~26.5%) (Figure 4F). Furthermore, we examined the effect of *OsCCR14* overexpression on leaf bleaching—a typical symptom of heat-induced damage (Figure 4E). A marked reduction in leaf bleaching area was observed in OE1, OE2, and OE3 (45.3%, 44.9%, and 46.1%, respectively), whereas the wild type exhibited a much higher bleaching area (~83.2%) (Figure 4E). Collectively, these results suggest that *OsCCR14* overexpression affects level of lignin and flavonols, thereby enhancing plant heat tolerance.

### 2.5. osccr14 Mutants Decrease Heat Tolerance at Seedling Stage

**Figure 5:**
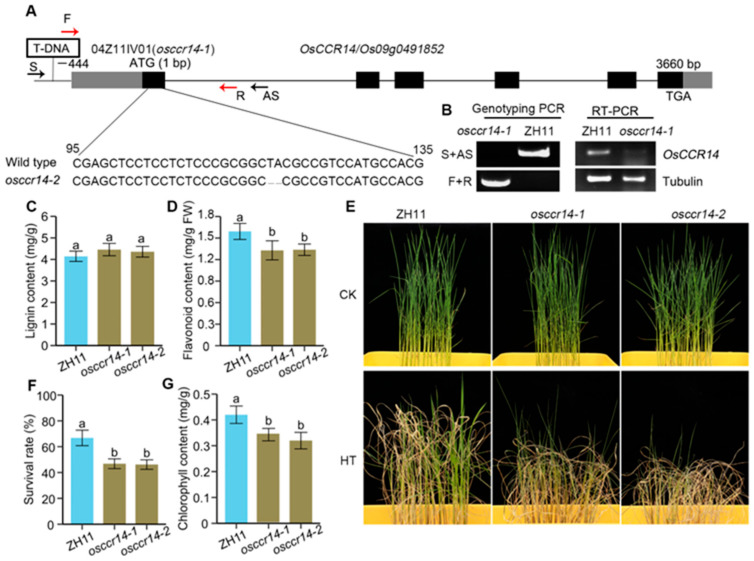
Identification of *osccr14* mutans and phenotypic analyses under high temperature. (**A**) Genomic structure of *OsCCR14* and schematic representation of the mutations in *osccr14-1* and *osccr14-2*. (**B**) Amplification of *OsCCR14* in genomic DNA or transcripts level from the ZH11, *osccr14-1* and *osccr14-2*. (**C**) Comparative analysis of lignin content in ZH11 and *osccr14* mutants. (**D**) Comparative analysis of flavonoid content in ZH11 and *osccr14* mutants. (**E**) Representative images of plant growth before and after heat stress treatment, respectively. (**F**) Plant survival rates of ZH11 and *osccr14* mutants under HT stress. (**G**) Chlorophyll content of ZH11 and *osccr14* mutants under HT stress. Values represented mean ± standard deviation of three independent biological replicates. Different letters above bars denoted significant differences (*p* ≤ 0.05, ANOVA with Duncan’s test). Colors are used solely to distinguish between different materials.

To further elucidate the biological function of OsCCR14, two *OsCCR14* knockout mutants were first identified and characterized. The first mutant, designated *osccr14-1* (accession number: 04Z11IV01), is a T-DNA insertion line isolated from the Rice Mutant Database (RMD). Genomic DNA amplification confirmed that the T-DNA was inserted into the untranslated region (UTR) 444 bp upstream of the ATG start codon (Figure 5A). RT-PCR analysis revealed a significant downregulation of *OsCCR14* transcript levels in OsCCR14-1 compared to the wild type (Figure 5B). The second knockout mutant, *osccr14-2*, was generated via CRISPR/Cas9-mediated genome editing. This mutant carries a 2-bp (“TA”) deletion at positions 117–118 of the first exon, which induces a severe frameshift mutation in the *OsCCR14* coding sequence, likely resulting in the loss of functional protein production (Figure 5A).

We next analyzed lignin and flavonoid contents in the two *osccr14* mutants and their wild-type background (ZH11) at the seedling stage (Figure 5C). Total lignin levels in *osccr14-1* and *osccr14-2* were comparable to those in ZH11. In contrast, a slight but detectable decrease in flavonoid content was observed in both mutants relative to the wild type (Figure 5D). To assess the role of *OsCCR14* in heat stress responses, we compared the heat tolerance of 16-day-old hydroponically grown *osccr14-1*, *osccr14-2* and ZH11 seedlings. Seedlings were exposed to 45 °C for 30 h to induce heat stress, followed by a 3-day recovery period at ambient temperature. Survival rates were then scored, and chlorophyll content was measured. As shown in Figure 5F, the survival rates of the mutants were significantly lower than that of ZH11: ~43.2% for *osccr14-1* and ~44.6% for *osccr14-2*, compared to ~68% for the wild type. Consistent with the survival phenotype, chlorophyll content was also reduced in both mutants after heat stress (Figure 5G). Collectively, these results indicate that OsCCR14 is involved in the regulation of heat tolerance in plants.

### 2.6. Overexpression OsCCR14-Enhanced Plant Thermotolerance

**Figure 6:**
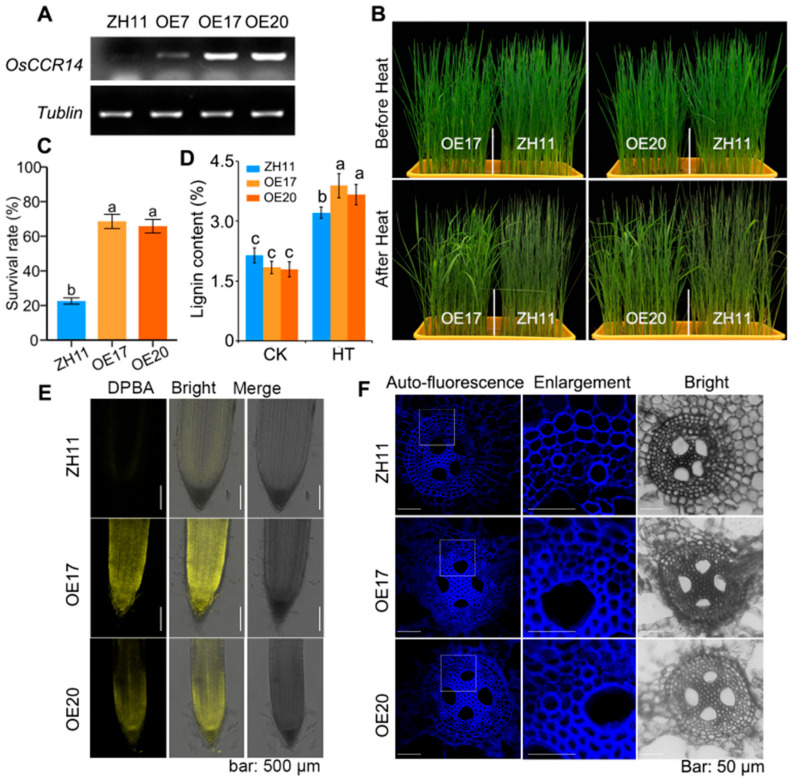
Phenotypic identification of *OsCCR14* overexpressing plants under high temperature. (**A**) RT-PCR analysis of OsCCR14 overexpressing rice lines. (**B**) Phenotype of OsCCR14 overexpressing rice lines under high temperature stress. (**C**) Survival rate of *OsCCR14* overexpressing rice lines under HT stress. (**D**) Analysis of total lignin content in OsCCR14 overexpressing rice plants before and after high temperature treatment. (**E**) DPBA staining of roots in OsCCR14 overexpressing rice lines. Values represented mean ± standard deviation of three independent biological replicates. (**F**) Lignin autofluorescence in root of OsCCR14 overexpressing rice lines under heat stress. Different letters above bars denoted significant differences (*p* ≤ 0.05, ANOVA with Duncan’s test).

To further validate the role of *OsCCR14* in heat tolerance, we generated transgenic rice plants overexpressing *OsCCR14*, using the enhanced CaMV *35S* promoter to drive transgene expression. RT-PCR analysis confirmed that *OsCCR14* transcript levels were significantly upregulated in all transgenic overexpression lines (Figure 6A). Consistent with the phenotype of *OsCCR14*-overexpressing Arabidopsis, overexpression of *OsCCR14* also increased flavonoid levels in rice roots. The DPBA-stained fluorescent signal (indicative of flavonol accumulation) in lines OE17 and OE20 was significantly stronger than that in the wild type (Figure 6E).

Next, we compared the heat tolerance of the transgenic rice overexpression lines and their wild-type background (ZH11) at the seedling stage. Under normal growth conditions, transgenic lines OE17 and OE20 exhibited growth comparable to that of ZH11. After exposure to heat stress, however, the transgenic lines displayed markedly improved leaf vitality, whereas the wild type showed more severe heat-induced damage (Figure 6B). The survival rates of OE17 and OE20 were ~42.5% and ~45.4%, respectively, significantly higher than that of the wild type (~8.6%) (Figure 6C). In addition, we determined the total lignin content of 2-week-old seedlings using the acetyl bromide method. Under normal growth conditions, there was no significant difference in lignin content between the overexpression lines and the wild type. However, high temperature treatment significantly increased lignin levels in all plants; notably, the overexpression lines exhibited a significantly higher lignin content than the wild type after heat stress (Figure 6D). Additionally, observations of lignin autofluorescence in root cross-sections revealed that secondary wall thickening was correspondingly reduced in root of OE17 and OE20 after heat stress (Figure 6F). Taken together, these results demonstrate that overexpression of *OsCCR14* enhances plant thermotolerance, and this enhancement is potentially mediated by increased lignin and flavonoid accumulation.

## 3. Discussion

The increasing frequency and severity of climate-related stresses pose a significant threat to global food security, driving the urgent need for strategies to enhance crop resilience [34]. As CCR catalyzes the first committed step of the lignin branch pathway, it plays a crucial role in secondary metabolism [35,36]. According to analysis of gene expression pattern, a significant increase in *OsCCR14* expression was detected following heat exposure. This observation aligns with previous findings demonstrating the involvement of CCRs in stress responses in various plant species. For instance, *zmccr1* mutants in maize exhibited altered lignin structure and changes in sclerenchyma cell wall development [37], highlighting the link between CCR activity and cell wall modifications under stress. In addition, the contrasting expression patterns elicited by ABA and SA, and the inhibitory effect of GA_3_ on *OsCCR14* expression, implied the intricate interplay of hormonal signaling pathways in regulating gene expression and stress tolerance. Exogenous SA has emerged as an effective strategy to mitigate heat stress (HT)-induced damage by upregulating the expression of genes in the phenylpropanoid pathway, leading to lignin accumulation [38]. In this study, we demonstrated that SA induces *OsCCR14* expression, implying that SA fine-tunes *OsCCR14* transcription to support lignin accumulation under heat stress, which aligns with previously findings. By contrast, the mild responsiveness of *OsCCR14* to ABA suggests this hormone does not act as a core regulator of *OsCCR14*-mediated lignin synthesis in rice. Notably, GA signaling is closely associated with the biosynthesis of secondary cell wall components: for example, HCT-RNAi alfalfa plants exhibit a dwarf phenotype accompanied by reduced GA levels, impaired GA perception, and decreased lignin content [39]. The observed repression of OsCCR14 by GA_3_ in our study may therefore be attributed to GA’s role in inhibiting *OsCCR14* to prevent excessive lignification—an outcome that could otherwise impair normal rice growth by restricting tissue flexibility and resource allocation to development.

The NWYCY motif is a signature of CCRs, distinguishing genuine CCRs from CCR-like proteins by its key role in CCR catalytic activity. However, OsCCR5 contains the NWYCY sequence but is classified as a CCR-like protein with no detectable CCR activity [25]. While *Selaginella moellendorffii* SmCCR1 and SmCCR2-1, with NWYCY substituted by NGYCL and EWYCL, respectively, have been verified as genuine CCRs in vitro [40]. Several researches also showed that the expression level of *CCR-like* gene in rice is consistent with the variation trend of lignin accumulation. A CCR-like gene *Snl6*, is required for pathogenesis-related gene expression and resistance to the bacterial pathogen, *Xanthomonas oryzae* pv. *oryzae* [41]. After *snl6* is mutated, the lignin content in the plant decreases significantly [41]. Another CCR-like gene, *OsCCRL1* involved in tapetum and pollen development. The *osccrl1* mutant exhibited reduced CCR enzyme activity, decreased lignin accumulation, delayed tapetum degradation, and disrupted phenylpropanoid metabolism [42]. In this study, we found that overexpression of *OsCCR14* led to increased lignin content and flavonoid levels in roots of transgenic Arabidopsis at normal growth condition (Figure 4). While the *osccr14* mutant exhibited a lignin level similar to that of the wild type at normal growth condition, but a clear increase after heat stress treatments (Figure 5). Notably, the unaltered lignin content in osccr14 mutants under normal conditions may stem from metabolic redundancy in lignin biosynthesis pathways, yet they showed reduced flavonoid levels and impaired heat tolerance. Similarly, a significantly enhanced lignin autofluorescence, indicative of secondary wall thickening, was observed in the roots of *OsCCR14*-overexpressing transgenic rice under heat stress (Figure 6). This lignin accumulation is consistently linked to higher survival rates in *OsCCR14*-overexpressing lines of both Arabidopsis and rice, and it underscores lignin might as the key driver of enhanced thermotolerance. Collectively, these results showed that OsCCR14 involved in lignification processes, especially in heat stress conditions.

Flavonoids, a group encompassing anthocyanins, flavonols, and proanthocyanidins (PAs), function as antioxidants to scavenge reactive oxygen species (ROS) and regulate ROS accumulation [43,44]. The flavonoid biosynthesis pathway belongs to one branch of the phenylpropanoid metabolic pathway, sharing the intermediate product coumaroyl-CoA with lignin biosynthesis pathway. Several key enzymes involved in the production of major flavonoid classes have been well characterized, including chalcone isomerase (CHI), flavanone 3-hydroxylase (F3H), dihydroflavonol 4-reductase (DFR), and anthocyanidin synthase (ANS) [45,46]. It was noticed that Borah et al. previously proposed that OsCCR14 might encode a dihydroflavonol-4-reductase, a function distinct from the canonical CCR activity [32]. In this study, we found that *OsCCR14* overexpression helps flavonoid levels accumulation in roots of both transgenic Arabidopsis seedlings and transgenic rice seedlings at normal growth conditions. We have not yet determined whether the OsCCR14 protein exhibits DFR activity. From the evolutionary and sequence analysis, OsCCR14 might present a high similarity to DFR protein (Figure 1). Given that *OsCCR14* is a *CCR-like* gene, it shares similarities with CCR proteins and, like DFR proteins, belongs to the short-chain dehydrogenase/reductase (SDR) superfamily; notably, both the CCR and DFR families are deeply rooted in the phylogeny of land plants [47]. One possible reason why overexpression of *OsCCR14* leads to an increase in both lignin and flavonoid levels, rather than a decrease in flavonoids. The possible explanation can be primarily attributed to the divergent substrate specificity of CCR-like protein. We hypothesize that OsCCR14 may catalyze certain hydroxycinnamoyl-CoA derivatives, which may alleviate feedback inhibition or competitive inhibition on the upstream shared pathway. This results in an upregulation of the total flux through the phenylpropanoid metabolic pathway, leading to increased synthesis of phenylalanine and coumaroyl-CoA in *OsCCR14*-overexpression transgenic plant. We therefore hypothesize that *OsCCR14* overexpression lines may enhance the level of lignin and flavonoids, reducing heat impair and thereby improving thermotolerance. In future studies, integrating transcriptomic and metabolomic analyses to identify key metabolites and elucidating the substrate specificity of OsCCR14 will deepen our understanding of the role of CCR-like proteins in heat stress responses.

## 4. Materials and Methods

### 4.1. Plant Materials

To generate *OsCCR14* overexpression plants, the coding sequence of *OsCCR14* (Os09g31514, LOC_09g0491852) was amplified from rice (*Oryza sativa* subsp. *japonica* ‘Nipponbare’). The *Japonica* cultivar Zhonghua11 (ZH11) was used as the non-transgenic control. The amplified *OsCCR14* coding sequence was cloned into rice transformation vector pGreenII carrying 35S promoter, and introduced into *Agrobacterium tumefaciens* strains GV3101 (pSoup) and then transformed into ZH11 or Arabidopsis Columbia ecotypes to generate transgenetic plants.

The gene editing sites and T-DNA insertion positions were finally determined by amplifying the target fragments, conducting sequencing, and performing sequence alignment. For the generation of knock-out (*osccr14-2*) mutants, we used the web-based tool CRISPR RGEN Tools (http://www.rgenome.net/, accessed on 8 June 2023). The method was performed as described previously [48]. *osccr14-1* was obtained from the Rice Mutant Database.

### 4.2. Stress Treatments

To investigate expression profile responses to abiotic stress and phytohormone treatments, seedlings of ZH11 were exposed to heat (HT; 42 °C) for varying durations (0, 3, 5, 12, 24, 36, and 48 h). For phytohormone treatments, seedlings were subjected to root immersion in solutions of different hormones, including 100 μM ABA, 10 mM SA, 10 mM gibberellic acid (GA_3_), and 10 mM ethylene (ETH), respectively. Tissue samples were collected during seedlings and flower stages. Samples collected at 0 h served as the control.

### 4.3. RNA Extraction, RT-PCR and qRT-PCR Analysis

To investigate the spatial and temporal expression patterns of *OsCCR14*, total RNA was isolated from shoots, roots, stem, leaf, spikers and anthers of 8–10 Nipponbare plants at the seedling and flowering stages. Approximately 100 mg of fresh tissue per sample was used, following the manufacturer’s instructions for Trizol reagents (Invitrogen, Wuhan, China).

RT–PCR was performed to determine the transcriptional levels of *OsCCR14* in transgenic lines, compared with wild-type plants, using the following cycling conditions: initial denaturation at 94 °C for 5 min, followed by 35 cycles of denaturation at 94 °C for 30 s, annealing at 58 °C for 30 s, and extension at 72 °C for 45 s, with a final extension at 72 °C for 10 min. qRT-PCR was carried out using a SYBR Premix Ex Taq II kit (Takara, Wuhan, China) on a QuantStudio 6 Flex Real-Time PCR System (Thermo Fisher Scientific, Shanghai, China). The amplification reactions were performed at 95 °C for 10 min, followed by 40 cycles of 95 °C for 5 s, 60 °C for 30 s, and 72 °C for 30 s. *18S rRNA* or *Tubulin* was used as an internal control for normalization as previously reported transcript, and two biological and three technical replicates were analyzed. Primers used are listed in Supplementary Table S1.

### 4.4. Subcellular Localization

The coding sequence of *OsCCR14* was fused to the *GFP* reporter gene under the control of the *35S* promoter, creating a *35S:OsCCR14-GFP* fusion construct using the vector pGreenII-GFP. Rice protoplasts were prepared and transfected following a modified protocol from Yoo et al. [49]. Following transfection, protoplasts were incubated in darkness at 25 °C for 16 h to allow for protein expression and subsequent subcellular targeting. To determine the subcellular localization of OsCCR14, an in vitro approach using transient expression in rice protoplasts was initially employed. GFP fluorescence was then visualized using a Leica TCSSP8 laser confocal microscope (Leica, Wetzlar, Germany), utilizing an excitation wavelength of 488 nm and an emission wavelength of 507 nm. Complementing the in vitro analysis, the 35S:OsCCR14-GFP construct was transformed into the Arabidopsis Columbia to assess OsCCR14 localization in vivo. Transgenic positive T_2_ plants were selected and confirmed to carry the transgene. GFP fluorescence was then observed in the root and leaf tissues of these transgenic plants to determine the protein’s cellular distribution.

### 4.5. Seedling Survival Rate Assessment

We evaluated the heat tolerance of transgenic, knockout, and non-transgenic plants at the vegetative stage, with experiments conducted in a greenhouse or controlled environment as follows. For *Arabidopsis thaliana*: wild-type (WT) Columbia ecotype and overexpression lines (OE1, OE2, OE3) were transplanted into soil pots (20 × 10 × 6 cm, 32 plants per pot) and grown for 2 weeks under greenhouse conditions (22 °C, 16 h light/8 h dark cycle). Seedlings were then exposed to heat stress at 42 °C for 48 h, followed by a 7-day recovery under normal growth conditions. For transgenic rice: Seeds of mutants, the wild-type ZH11, and T_3_ homozygous overexpression (OE) lines were first soaked in prochloraz for 2 h, rinsed thoroughly with distilled water, and soaked overnight. The next day, seeds were germinated in a moist environment at 30 °C. Germinated seeds were transferred to bottomless 96-well plates and hydroponically cultured in 1× nutrient solution in a light incubator (26 °C, 16:8 light–dark photoperiod). At the two-leaf-one-heart stage, seedlings were subjected to 45 °C high-temperature stress in a growth chamber until obvious wilting occurred, followed by a 3-day recovery at 26 °C under normal conditions.

### 4.6. Ratio of Leaf White Area Assessment

Leaf bleaching was quantified by measuring the ratio of leaf white area (%) in *Arabidopsis thaliana* (Columbia, OE1, OE2, OE3). Digital photographs of leaves under heat stress and control were captured (Leica, Wetzlar, Germany) and analyzed using ImageJ software (version 1.53j). For each leaf, the total leaf area and bleached area were manually delineated using the freehand selection tool in ImageJ. The bleaching rate was calculated as the ratio of bleached area to total leaf area. Experiments included at least three biological replicates, each consisting of 10 plants.

### 4.7. Determination of Lignin and Flavonoid Level

Lignin content was determined via the thioglycolic acid method, as previously described by Ding et al. [48], using 0.1 g of plant (whole seedlings) powder per sample and a standard curve (0–0.5 mg/mL, AccuStandard, CAS#9005-53-2) for quantification. The cell wall fraction of the samples was prepared by removing interfering compounds through sequential extractions with phosphate buffer, Triton X-100, and NaCl in acetone. For observing lignin autofluorescence in root cross-sections, young root tissues from three-week-old rice plants were fixed in FAA for 48 h. Fixed tissues were gradually dehydrated using a graded ethanol series to remove water, then treated with a clearing agent to eliminate ethanol and render the tissues permeable to paraffin. After paraffin infiltration and embedding, 10 μm-thick sections were cut using an RM2235 microtome (Leica, Wetzlar, Germany). Lignin autofluorescence in these sections was observed under 405 nm UV light using a fluorescence microscope (Leica TCS SP5, Wetzlar, Germany).

The total flavonoid content was determined via the aluminum chloride colorimetric assay, as previously described by Cai et al. [31], using 0.1 g of plant powder per sample and a rutin standard solution (0–1.0 mg/mL, Solarbio, SR8250) for quantification. To assess flavonol accumulation in roots, in situ staining with diphenylboric acid 2-aminoethyl ester (DPBA) was conducted. Roots of 10-day-old seedlings were stained with an ethanol solution containing 0.25% (*w*/*v*) DPBA and 0.01% (*v*/*v*) Triton X-100 until a saturated signal was observed (for at least 1.5 h). The resulting fluorescence was visualized using a laser confocal microscope (Leica, Wetzlar, Germany) with an excitation wavelength of 458 nm.

### 4.8. Statistical Analysis

Using R (version 4.5), one-way ANOVA was performed to determine mean differences between groups. Statistically significant differences (*p* ≤ 0.05) were then evaluated with Duncan’s multiple comparison test, implemented through the duncan.test function in the agricolae package (version 1.3-7).

## 5. Conclusions

In summary, we identified that *OsCCR14*, a CCR-like gene, plays a key role in regulating lignin and flavonoid biosynthesis, thereby enhancing plant heat tolerance. While *OsCCR14* expression correlated with lignin content, its downregulation only slightly reduced lignin levels. In contrast, *OsCCR14* overexpression significantly increased lignin and flavonoid accumulation, particularly under heat stress. Thus, we infer that *OsCCR14* improves rice heat tolerance by promoting root lignification and flavonoid accumulation, which enhance structural stability and antioxidant defense capacity, respectively. Our findings provide new insights into the role of CCR-like genes in rice heat tolerance.

## Data Availability

All relevant data can be found and available within the manuscript.

## Supplementary Materials

The following supporting information can be downloaded at: https://www.mdpi.com/article/10.3390/plants14233626/s1, Table S1: Primers used in this study.
